# Funding for research on cryptococcal disease: an analysis based on the G-finder report

**DOI:** 10.1186/s43008-023-00133-6

**Published:** 2024-03-02

**Authors:** Iraine Duarte, Marcio L. Rodrigues

**Affiliations:** 1 Instituto Carlos Chagas, Fundação Oswaldo Cruz (Fiocruz), Rua Professor Algacyr Munhoz Mader 3775- CIC, Curitiba, PR 81350-010 Brazil; 2 Instituto de Microbiologia Paulo de Góes (IMPG), Universidade Federal do Rio de Janeiro. Cidade Universitária, Centro de Ciências da Saúde., Rio de Janeiro, RJ 21941-902 Brazil

**Keywords:** Neglected mycoses, Funding, Cryptococcal meningitis

## Abstract

Members of the genus *Cryptococcus* are the causative agents of cryptococcal meningitis, a disease mainly associated with HIV-induced immunosuppression. Patients with cryptococcal meningitis are at a serious risk of death. Most patients suffering from cryptococcosis belong to neglected populations. With reduced support for research, new therapies are unlikely to emerge. In this essay, we used the Policy Cures/G-finder platform as a reference database for funding research on cryptococcal disease. Funding for cryptococcal research started being tracked by G-finder in 2013 and has continued to appear in the annual reports ever since. In total, 15 institutions were reported as major funders for research on cryptococcal disease over the years. The US National Institutes of Health (NIH) was the main funder, followed by the UK's Wellcome Trust. The annual analysis suggested slow yearly growth in funding from 2013 to 2021. The development of new tools to prevent and fight cryptococcal disease is urgent but requires improved funding.

## INTRODUCTION

Diseases of neglected populations represent a devastating obstacle to health and remain a serious impediment to poverty reduction and socioeconomic development, contributing to a cycle of penury through their effects on health and well-being (Bangert et al. 2017; Engels and Zhou 2020). Cases of invasive fungal diseases are on the rise as the at-risk population continues to expand (Firacative et al. 2018). This increase can be attributed to various factors, including advancements in modern medicine and the greater accessibility of therapies and interventions that compromise the immune system (World Health Organization 2022).

The COVID-19 pandemic has been associated with a rise in the incidence of comorbid invasive fungal infections (Hoenigl et al. 2022; Regalla et al. 2022). The underrecognized and emerging global health threat posed by invasive fungal diseases is exacerbated by the rapid emergence of antifungal resistance and, in many settings, limited access to quality diagnostics and treatment (Rodrigues and Nosanchuk 2020). In recent years, numerous proposals have been put forward to mitigate the fatalities caused by fungal diseases (Mapook et al. 2022). However, despite these efforts, the detrimental effects of these diseases on public health and the economy persist (Rodrigues and Nosanchuk 2023).

The genus *Cryptococcus* includes several species found in different environments (Denham et al. 2022), of which seven are typical human pathogens (Hagen et al. 2017; Li et al. 2020), and *C. neoformans* is the main one that causes disease in association with HIV-mediated immunosuppression (Godinho et al. 2017). Recently, the WHO classified *C. neoformans* as a critical fungal pathogen (World Health Organization 2022). Cryptococcal meningitis occurs when a lung infection spreads to the brain, causing a highly lethal disease when early diagnosis and adequate treatment are not provided (Li et al. 2020; Okwir et al. 2022). The fight against cryptococcal meningitis is impaired by problems with antifungal therapy (Rodrigues 2018). In addition to the limited number of classes of antifungal drugs, less toxic antifungals are costly, resulting in reduced availability in countries where cryptococcal meningitis is more prevalent (Boyer-Chammard et al. 2019; Loyse et al. 2019; Rathore et al. 2022) For instance, liposomal amphotericin B (AmB) in combination with flucytosine can effectively treat cryptococcal meningitis, but these drugs are poorly suited for use in less developed countries (Larson et al. 2021). Liposomal AmB is expensive and requires hospital administration (Jarvis et al. 2022), while flucytosine requires careful blood monitoring (Molloy et al. 2018). Consequently, cryptococcal meningitis is typically treated with fluconazole in less developed countries, which is only partially effective (Bermas and Geddes-McAlister 2020; Sloan and Parris 2014). Prolonged use of fluconazole has the potential to exert selection pressure in favor of fluconazole-resistant strains (Bongomin et al. 2018; Lahiri and Chandrashekar 2022), which is a serious problem in *Cryptococcus* isolates taken from patients experiencing relapses (Dembelu and Woseneleh 2021; Wykowski et al. 2020).

The combination of high mortality, toxicity, and the cost of therapy highlight the urgent need for effective and affordable medicines suitable for use in low-resource settings (Mourad and Perfect 2018; Patel et al. 2018). To reduce mortality, the rational use of existing tools is undoubtedly important, but stimulating research, technological development, and innovation is the most effective way to address this complex scenario (Rodrigues and Nosanchuk 2020). We reviewed the findings provided by the Policy Cures/G-finder platform to map funding for research on cryptococcal disease over the past decade (Policy Cures Research 2022). Our analysis reveals a concerning picture that suggests the necessity of improved funding for cryptococcal meningitis and fungal infections in general to generate qualified knowledge and innovative tools to combat this major public health problem.

## THE G-FINDER FUNDING DATABASE

Policy Cures is an independent group that provides information that is potentially helpful for decision-making analyses and strategic plans to stakeholders involved in the development of new drugs for diseases of neglected populations (Policy Cures Research 2022). They provide governments, funders, and organizations with the necessary information to foster research and development (R&D) policies and funding decisions for diseases prevalent in the less developed world. Policy Cures has reported global investments in neglected disease research and development through G-Finder reports. G-Finder serves as a data source that provides objective and previously unavailable information on investment status, trends, and patterns.

### Tracking of funding

**Fig. 1:**
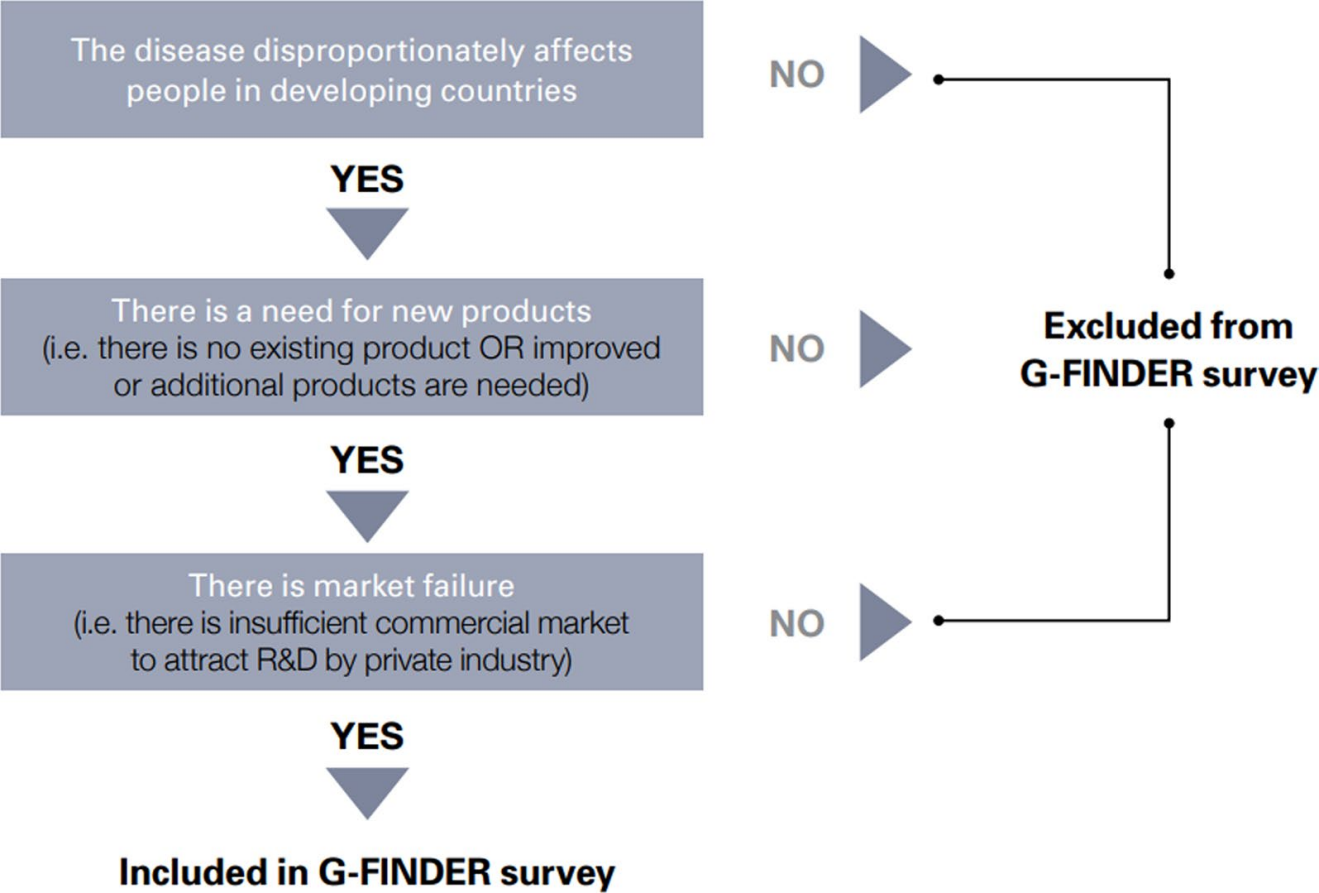
Identifying neglected diseases according to G-Finder (Policy Cures Research 2022)

The methodology of the G-Finder data portal offers users background information on the presented data, its scope, and the methodology employed. Funding data are adjusted for inflation and converted to US dollars to account for the impacts of inflation and fluctuations in exchange rates (Policy Cures Research 2022). The neglected diseases covered by G-Finder research are determined in consultation with an Advisory Committee comprising a diverse group of international experts in neglected diseases and product development. This determination is based on a three-phase approach, as depicted in Fig. 1.

### Research stage

Funding included in G-Finder covers various scopes of R&D: basic research, drugs, vaccines, biologics, diagnostics, microbicides, and vector control products. These activities are further categorized into two main groups: basic and early-stage research and clinical, or field development and post-registration studies (Policy Cures Research 2022). Basic and early-stage research encompasses fundamental research and discovery, as well as preclinical development. Clinical or field development and post-registration studies include baseline epidemiology in preparation for product trials, clinical development, and field evaluation, post-registration studies of new products, and operational research for diagnostics. It is important to note that not all product areas are included for all diseases within G-Finder. The inclusion of specific product areas may depend on additional predetermined conditions.

### Investments for neglected diseases according to G-finder

G-Finder classifies neglected diseases into three tiers. Top tier diseases, including HIV/AIDS, malaria, and tuberculosis, receive the majority of global R&D funding for neglected diseases. The 'second tier' diseases, such as diarrheal diseases, kinetoplastids, bacterial pneumonia and meningitis, helminth infections, dengue, and *Salmonella* infections, receive between 1.0% and 10% of the total funding (Policy Cures Research 2022). The 'third tier' diseases, such as leprosy, Buruli ulcer, trachoma, and rheumatic fever, receive less than 0.5% each of the global funding for neglected disease R&D, making them the most neglected diseases. In the G-Finder 2014 Report, a change was made following a review by the new Advisory Committee (AC). The survey was expanded to include three additional diseases: Hepatitis C (genotype 4), leptospirosis, and cryptococcal meningitis. Due to the limited number of grants from a few funders each year, trend analysis between 2007 and 2012 was not conducted for cryptococcal meningitis, and it did not appear in the early G-Finder reports (Policy Cures Research 2022).

**Fig. 2:**
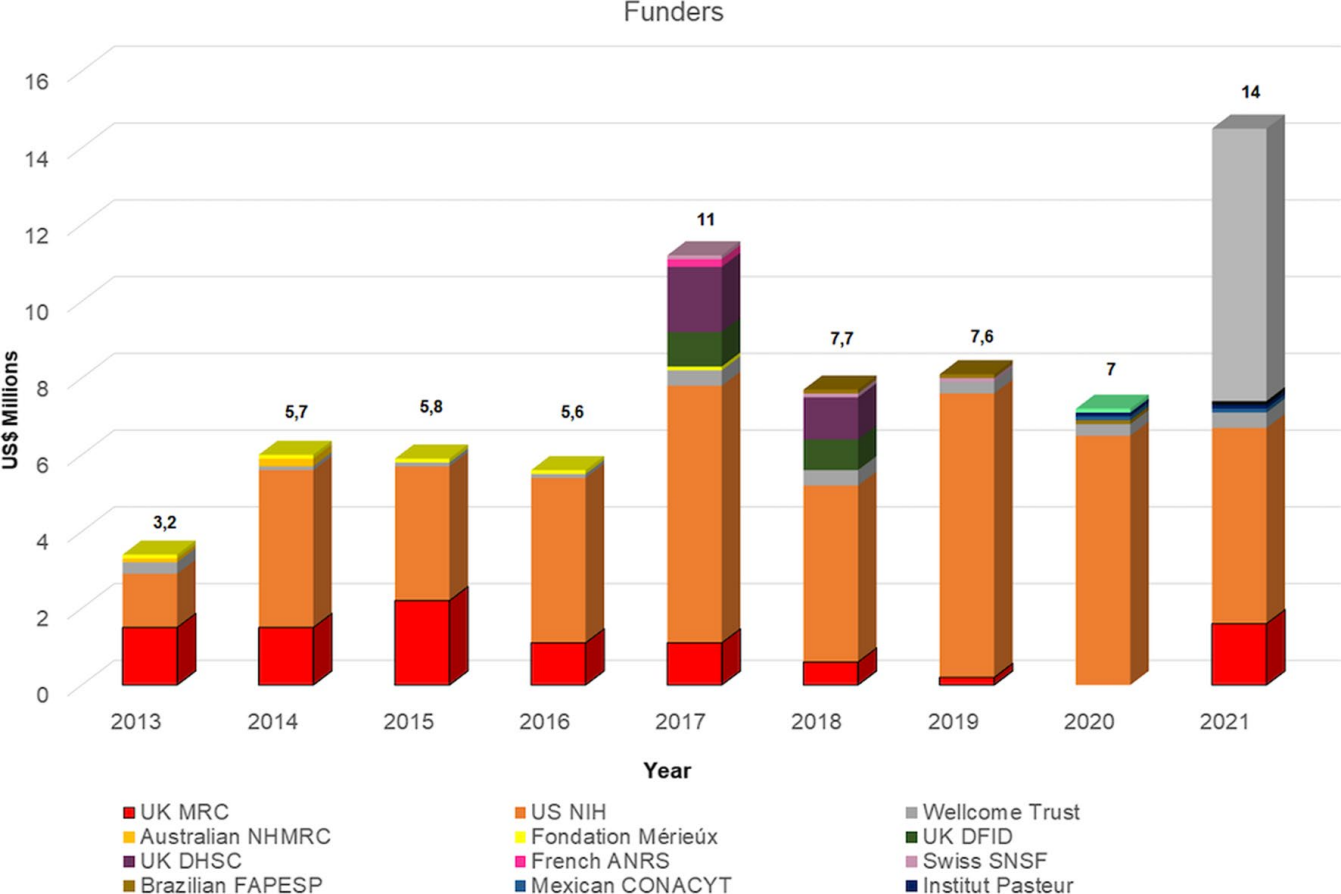
Funding Agencies for Cryptococcal Research from 2013 to 2021 in USD (millions), as per G-Finder (Policy Cures Research 2022)

Cryptococcal meningitis was only tracked by G-Finder from 2013, and has been classified as one of the most poorly funded diseases, referred to as a "third-tier disease", and it remains one of the most underfunded of the diseases in the most recent report. In total, major funding for cryptococcal meningitis was provided by 15 institutions over the years (Fig. 2), but only two have consistently funded the disease from the 2014 G-Finder report to the latest report in 2022. The US National Institutes of Health (NIH) was initially ranked as the second-largest investor, representing 42% of the total funding, and subsequently became the main funder in the following years, reaching 91% in 2020. The Wellcome Trust is the second recurring investor.

In 2017, eight institutions were reported to provide funding, which is twice as many as in 2016. There was an unexpectedly large investment of nearly $11 million in 2017, representing the largest annual investment for cryptococcal research up to that date. However, there was a significant decline in funding from 2018 to 2020. Nevertheless, funding remained approximately $2 million higher than in pre-2017 levels, despite the impact of COVID-19. It is worth noting that funding per agency is a naturally variable activity that can be explained by multiple reasons, including policy changes, project conclusion, and a lack of robust competitive applications.

Even considering these variables, it was interesting to note that in 2020, despite three new funders coming on board for cryptococcal research, there were no reported contributions from two other previous funders. The UK Medical Research Council (MRC), which had been the most noticeable funder in 2013, became the second-largest for the following five consecutive years, but with funding gradually declining. This reduction in UK MRC funding was likely a result of the completion of long-term funding streams, including those for high-dose AmBisome Phase III trials and sertraline.

Almost all of the reported investment in cryptococcal meningitis R&D, since its inclusion in G-Finder from 2013 to 2020, originated from the public sector, constituting 98% of the reported investment; the remainder came from philanthropic organizations. The year 2021 marked an exception, as a significant investment was reported from the private sector (Policy Cures Research 2022). In fact, funding for R&D of drugs and biologics for cryptococcal meningitis reached a record high in 2021, totaling $14 million. This marked a significant increase of $7 million (97%), compared to 2020. The surge in funding was primarily attributed to a single small and medium-sized enterprise (SME) that reported funding for the first time that year, indicating a possible industry interest in cryptococcal R&D. While private sector funding saw a notable influx, contributions from ongoing survey participants remained largely unchanged. Funding from the NIH, which had been the top funder in previous years, experienced a substantial decrease of $1.7 million (–25%). However, this decline was mostly offset by the resumption of funding from the UK MRC, with an increase of $1.6 million.

Since 2013, resources for cryptococcal research have primarily been allocated to projects involving drug development as the result of research activity, as highlighted within the scope of G-Finder. The remaining resources have been dedicated to clinical development. In 2018, the G-Finder scope for cryptococcal research was expanded to include funding for biological tools, which previously encompassed therapeutic vaccines, monoclonal antibodies, and preventive vaccines. However, the reported funding values were relatively low and only representative of 2020, totaling US$ 0.13 million, approximately 2%. In 2021, the entry of the private sector resulted in an increase in funding to US$0.74 million (5%).

## CONCLUSIONS

The funding landscape for cryptococcal research has been uncertain, characterized by fluctuations and limited growth in funding over the years. While there was slow annual growth in funding from 2013 to 2021, notable exceptions included a significant increase in 2017 and another in 2021. The overall funding value has, nevertheless, remained relatively stable, with few new funders entering the scene. This instability in future funding projections raises concerns, particularly given that cryptococcal meningitis ranks among the most underfunded conditions within fatal diseases. To address the urgent need to eliminate cryptococcal meningitis-related deaths by 2030 (Shroufi et al. 2021), there is a pressing demand for increased investment in cryptococcal diagnostics, meningitis treatments, and the implementation of preventive screening measures (Rajasingham et al. 2022; Shroufi et al. 2021).

To bridge the funding gap and propel research across diverse facets of cryptococcal disease, the imperative lies in augmenting resources, and engaging joint activities ideally involving multiple organizations. This strategic move would facilitate comprehensive research encompassing various dimensions of the ailment, such as clinical manifestations, virulence factors, susceptibility to antifungals, and diagnostic attributes. These investigations would pave the way for the execution of global surveillance initiatives. The urgency of addressing these challenges cannot be overstated, as these are necessary to increase access to early diagnosis and suitable antifungal interventions, thereby mitigating mortality rates associated with cryptococcal meningitis. In this endeavor, collaboration among researchers, public health authorities, funding entities, and other stakeholders is pivotal to forge ahead in the battle against this overlooked disease.

## Data Availability

The datasets during and/or analyzed during the current study are available from the corresponding author on reasonable request.

## Abbreviations

COVID-19: Corona Virus Disease 2019 caused by SARS-CoV-2
MRC: Medical Research Council
NIH: National Institutes of Health
R&D: Research and development
SME: Small and medium-sized enterprise
UK: United Kingdom
US: United States of America
WHO: World Health Organization

## Declarations

### Adherence to national and international regulations

This manuscript and its content follow all national and international regulations relevant to article writing, interpretation and publication.
